# Successful Treatment of Linear IgA Disease and Ulcerative Colitis With Sulfasalazine

**DOI:** 10.7759/cureus.37210

**Published:** 2023-04-06

**Authors:** Drew Fletcher, Sagar Patel, Kiran Motaparthi

**Affiliations:** 1 Department of Dermatology, University of Florida, Gainesville, USA

**Keywords:** sulfapyridine, dapsone, sulfasalazine, ulcerative colitis (uc), subepidermal blistering disorders, linear iga bullous dermatosis, linear iga disease

## Abstract

Linear IgA disease (LAD) is an uncommon autoimmune blistering disease that has been associated with medications, malignancy, and other autoimmune diseases, such as ulcerative colitis (UC). In this case report, a patient with a history of UC developed characteristic LAD lesions. While dapsone is considered first-line therapy for LAD, the treatment team opted for an underutilized, plausibly less toxic, and more simplified treatment regimen with sulfasalazine, successfully utilizing the two distinct actions of sulfasalazine’s components - sulfapyridine and 5-aminosalicylate (5-ASA) - to concurrently treat both the LAD and UC symptoms. The authors discuss the pathophysiology of LAD and UC and expound on the mechanistic theory of their association. Additionally, the pharmacodynamics of sulfasalazine and considerations of its side effect profile are examined.

## Introduction

Linear IgA disease (LAD) is a rare, acquired autoimmune blistering disease with childhood and adult variants. LAD presents as annular or polycyclic erythematous plaques with tense vesicles and bullae on the periphery of the lesions, which may form a characteristic “crown of jewels” pattern. In adults, these lesions are primarily distributed on the trunk, limbs, and head with mucosal involvement seen in about 50% of cases [1]. LAD is characterized by a subepidermal blister with a neutrophil-predominant inflammatory infiltrate on histopathology and linear IgA1 deposits at the basement membrane zone on direct immunofluorescence (DIF) [2].

Medications, malignancy, and several autoimmune diseases have been described in association with LAD. Multiple retrospective studies have demonstrated an association between LAD and ulcerative colitis (UC), with UC preceding the onset of LAD in most patients [3]. Dapsone, a sulfone, is the first-line treatment for LAD. Sulfonamides, such as sulfapyridine, have also been used with success as a second-line treatment for LAD [1]. Sulfasalazine, a medication consisting of 5-aminosalicylate (5-ASA) and sulfapyridine linked by a diazo bond, is often used in the treatment of UC [4]. Herein, we describe LAD arising in association with UC and their successful treatment with sulfasalazine.

## Case presentation

**Figure 1:**
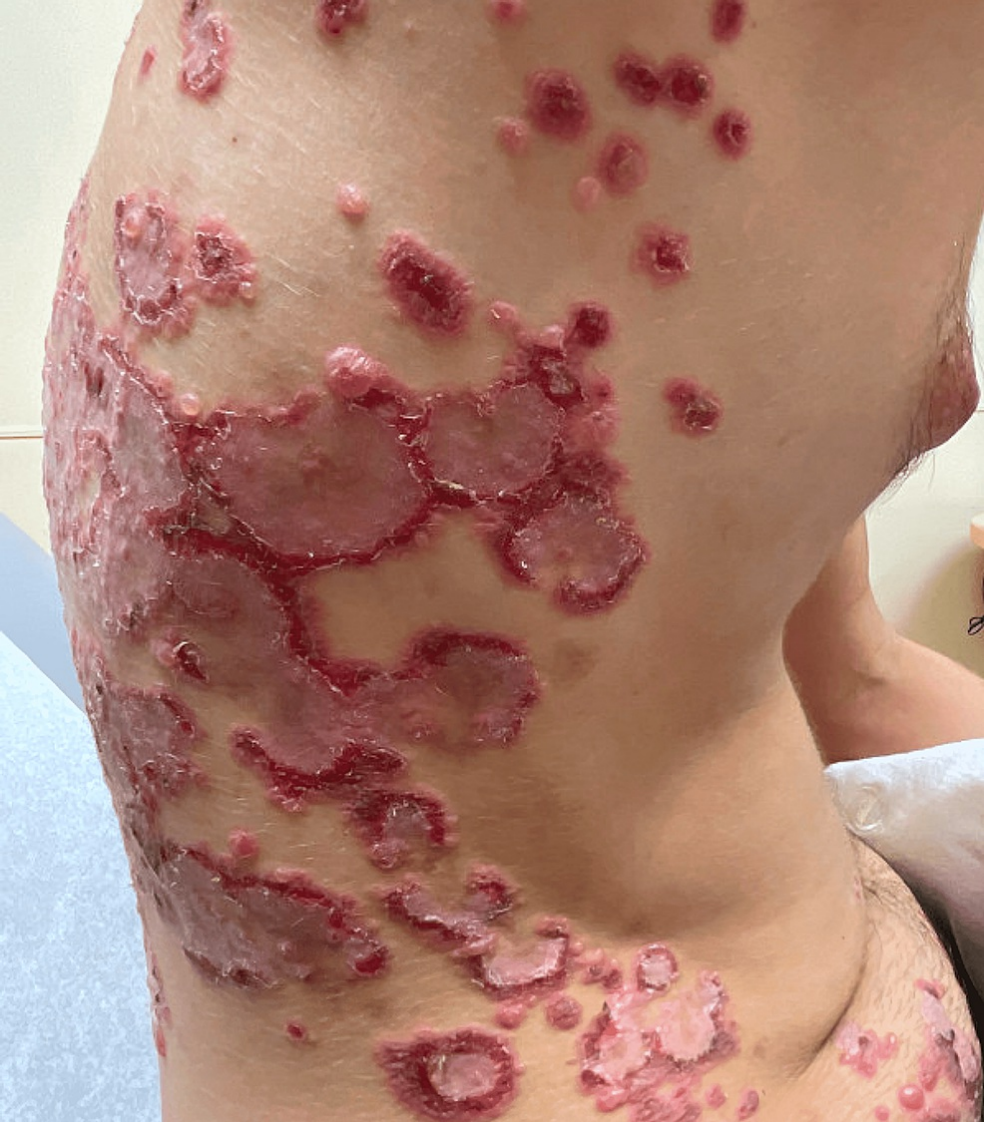
Tense vesicles and bullae coalescing in polycyclic configurations on the trunk

**Figure 2:**
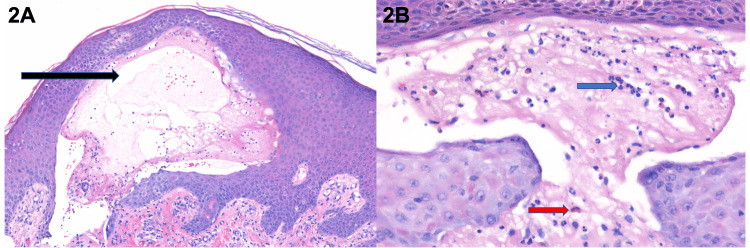
Histopathology of linear IgA disease 2A: Subepidermal blister (black arrow) in linear IgA disease (H&E, 150× magnification) 2B: Neutrophils (blue arrow) and eosinophils (red arrow) within the subepidermal blister cavity (H&E, 400× magnification)

A 19-year-old man with a 10-month history of UC and iron deficiency anemia presented with pruritic eruption. The eruption coincided with the onset of symptoms due to UC, including worsening diarrhea, severe abdominal cramping pain, and eventually rectal bleeding. Colonoscopy revealed severe pancolitis, and histopathology was consistent with UC. Tense vesicles and bullae with yellow serum, some of which coalesced to form polycyclic configurations, were observed on the flexures, trunk, and extremities (Figure 1). The differential diagnosis included dermatitis herpetiformis, bullous pemphigoid, pemphigus vulgaris, erythema multiforme, epidermolysis bullosa acquisita, and Stevens-Johnson syndrome. Histopathology demonstrated a subepidermal blister with neutrophils (Figures 2A, 2B). DIF demonstrated thick linear IgA deposition along the epidermal basement membrane zone and was negative for other immunoreactants.

**Figure 3:**
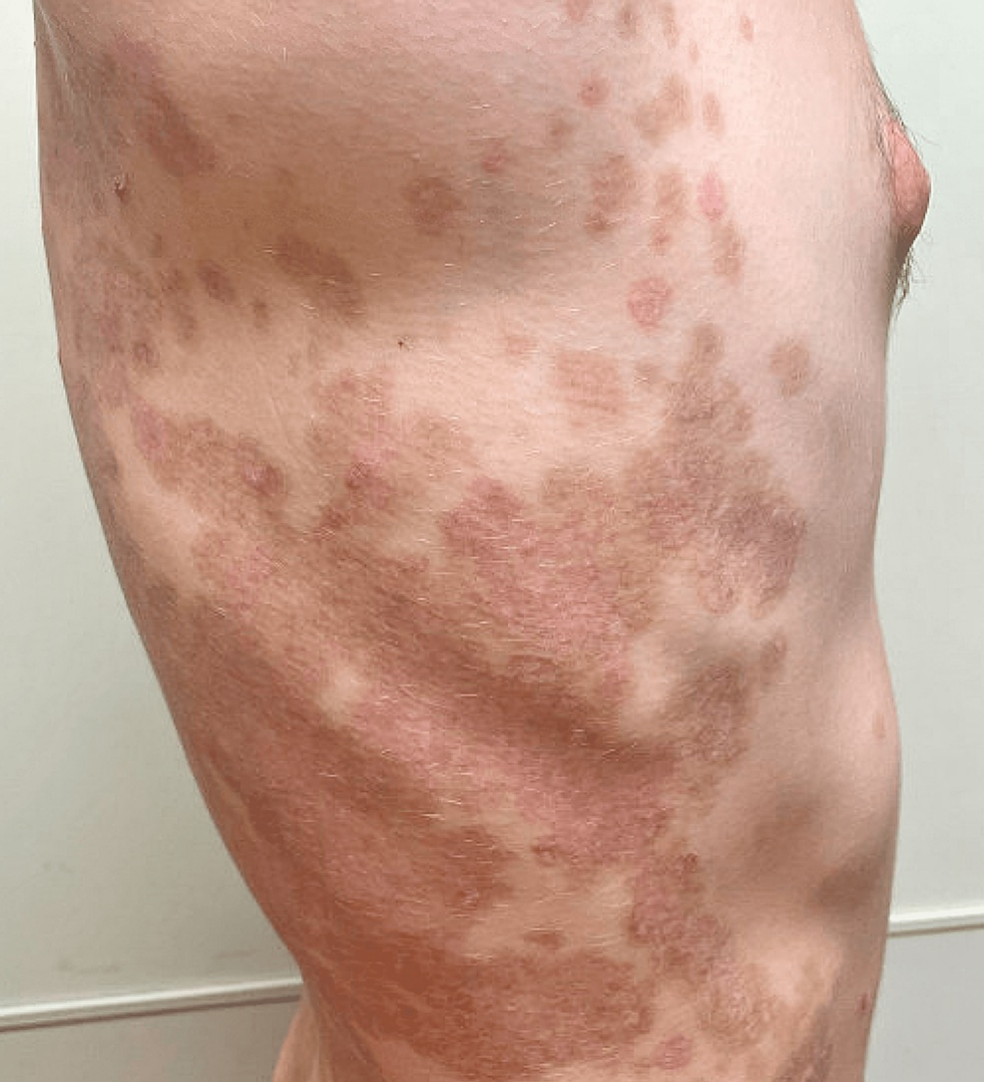
Resolution with post-inflammatory hyperpigmentation following treatment with sulfasalazine

Treatment with prednisone 30 mg daily and mesalamine 1.2 g twice daily was initiated, which improved the findings of LAD and the symptoms of UC, respectively; however, both conditions flared as prednisone was tapered. In lieu of dapsone, treatment with sulfasalazine 1 g three times daily was initiated, and mesalamine was discontinued. Prednisone was tapered over several weeks and then discontinued. The patient tolerated the sulfasalazine treatment well with no reported adverse effects. At the follow-up, two months later, the patient had been off prednisone for six weeks, and the findings of LAD had resolved with post-inflammatory changes (Figure 3).

## Discussion

The association between UC and LAD has been established, but the mechanism of this association has not yet been fully explained. The pathogenic IgA class autoantibodies in the sera of patients with LAD bind to LABD97 (97 kDa) or LAD-1 (120 kDa), both of which represent extracellular processed domains of BP180 (bullous pemphigoid antigen 180) [5]. High levels of BP180 RNA expression have been observed in multiple epithelia, including the colon [6]. In UC, B cells in colonic lymphoid tissue secrete relatively high levels of IgA1 with low levels of total IgA compared to control tissue. IgA1 is the major antibody implicated in LAD [7]. In UC, chronic mucosal inflammation leads to defects in the integrity of mucous and epithelial barriers, resulting in erosions and ulcers of the rectum and colon [8]. Collectively, these findings suggest that the breakdown of the colonic mucosa and abnormal antigen processing leads to the subsequent production of pathogenic IgA1 autoantibodies directed against LABD97 or LAD-1 in patients with UC. These antibodies deposit in the basement membrane zone of susceptible individuals, resulting in manifestations of LAD in some patients with UC. This mechanistic theory also explains the temporality of the association between UC and LAD, with the former usually preceding the latter.

Sulfasalazine, which is formed by 5-ASA and sulfapyridine linked by a diazo bond (N=N), is often used in the treatment of UC. In the colon, bacterial azo-reducing enzymes cleave this bond, and the 5-ASA moiety can exert its therapeutic effect in the gastrointestinal tract, while the sulfapyridine is absorbed systemically [4]. The molecular structures of dapsone and sulfapyridine are similar, and they exert their clinical effect through their aromatic amine functional groups. They are antimicrobials that both function by impeding microbial folate synthesis through competitive inhibition with para-aminobenzoic acid (PABA), an intermediate in the folate synthesis pathway. However, in inflammatory disorders including neutrophilic dermatoses, dapsone and sulfapyridine inhibit the cytotoxic myeloperoxidase-peroxide-halide system, a component of the neutrophil respiratory burst, and suppress neutrophil chemotaxis [2].

The systemic adverse effects of sulfasalazine are largely the same as those of dapsone and are believed to derive from its sulfapyridine component, rather than the 5-ASA, which has minimal absorption and is excreted in the feces. As with dapsone, sulfapyridine is oxidized to a hydroxylamine metabolite that causes pharmacologic hemolysis (particularly in patients with glucose-6-phosphate dehydrogenase deficiency), methemoglobinemia, and idiosyncratic agranulocytosis [2]. Dapsone hydroxylamine leads to a greater degree of cytotoxicity and methemoglobinemia than does sulfapyridine hydroxylamine. This enhanced tolerability of sulfapyridine compared to dapsone may reflect the shorter half-life of sulfapyridine hydroxylamine compared to that of dapsone hydroxylamine (eight versus 37 minutes, respectively) [4].

There are few descriptions of the use of sulfasalazine for LAD. Sulfasalazine has been used due to regional differences in the availability of dapsone [9] and in the context of primary sclerosing cholangitis and hepatitis, relative contraindications for dapsone [10]. Sulfasalazine may be underrecognized as a therapeutic option for subepidermal blistering disorders with neutrophils including LAD. In patients with UC and LAD, sulfasalazine may represent a convenient therapy for both diseases.

## Conclusions

This case report highlights the association between UC and LAD and the successful treatment of both diseases with a single agent known as sulfasalazine. The patient presented with symptoms of UC followed by symptoms of LAD, and this pattern of temporality can be explained by the breakdown of colonic mucosa in UC and an abnormal immune response to exposed antigens, leading to the pathogenic IgA1 autoantibodies found in the sera of patients with LAD. Furthermore, sulfasalazine’s two component moieties have separate clinical effects that concurrently target UC and LAD. Once cleaved from each other, the 5-ASA component acts locally in the gastrointestinal tract to reduce inflammation in UC, while the sulfapyridine component is absorbed systemically and suppresses the neutrophil action involved in LAD.
